# The prevalence of disrespect and abuse during facility-based maternity care in Malawi: evidence from direct observations of labor and delivery

**DOI:** 10.1186/s12978-017-0370-x

**Published:** 2017-09-06

**Authors:** Reena Sethi, Shivam Gupta, Lolade Oseni, Angella Mtimuni, Tambudzai Rashidi, Fannie Kachale

**Affiliations:** 1 Jhpiego, 1776 Massachusetts Ave NW, Washington, DC USA; 2 Department of International Health, The Johns Hopkins Bloomberg School of Public Health, Baltimore, USA; 3 Jhpiego, 1615 Thames Street, Baltimore, MD USA; 4 USAID, Lilongwe, Malawi; 5 Jhpiego, Lilongwe, Malawi; 6 Reproductive Health Directorate, Ministry of Health, Lilongwe, Malawi

**Keywords:** Respectful maternity care, Disrespect and abuse, Malawi, Direct observations

## Abstract

**Background:**

There is increasing evidence throughout the world that the negative treatment of pregnant women during labor and delivery can be a barrier to seeking skilled maternity care. At this time, there has been little quantitative evidence published on disrespect and abuse (D&A) in Malawi. The objective of this research is to describe the prevalence of disrespect and abuse during labor and delivery through the secondary analysis of direct clinical observations and to describe the association between the observation of D&A items with the place of delivery and client background characteristics.

**Methods:**

As part of the evaluation of the Helping Babies Breathe intervention, direct observations of labor and delivery were conducted in August 2013 from 27 out of the 28 districts in Malawi. Frequencies of disrespect and abuse items organized around the Bowser and Hill categories of disrespect and abuse and presented in the White Ribbon Alliance’s Universal Rights of Childbearing Women Framework were calculated. Bivariate analysis was done to assess the association between selected client background characteristics and the place of delivery with the disrespect and use during childbirth.

**Results:**

A total of 2109 observations were made across 40 facilities (12 health centers and 28 hospitals) in Malawi. The results showed that while women were frequently greeted respectfully (13.9% were not), they were often not encouraged to ask the health provider questions (73.1%), were not given privacy (58.2%) and were not encouraged to have a support person present with them (83.2%). Results from the bivariate analysis did not show a consistent relationship between place of delivery and D&A items, where the odds of being shouted at was lower in a health center when compared to a hospital (OR: 0.19; CI: 0.59–0.62) while there was a higher odds of clients not being asked if they have any concerns if they were in a health center when compared to a hospital (OR: 2.40; CI: 1.06–5.44). Women who were HIV+ had significantly lower odds of not having audio and visual privacy (OR: 0.34, CI: 0.12–0.97), of not being asked about her preferred delivery position (OR: 0.17, CI: 0.05–0.65) and of not being asked if she has any other problems she is concerned about (OR 0.38, CI:0.15–0.96).

**Conclusion:**

This study is among the first to quantify the prevalence of disrespect and abuse during labor and delivery in Malawi through direct clinical observations. Measurement of the poor treatment of women during childbirth is essential for understanding the scope of the problem and how to address this issue.

## Plain English summary

In recent years, more information has become available about how women are treated during labor and delivery. This information is important because there is existing evidence that if women are disrespected and/or abused during delivery care, they may not be as likely to seek this type of care. The purpose of this research was to provide the results about whether women were disrespected and abused using observations of labor and delivery in Malawi.

The results showed that most women were greeted respectfully but were often not encouraged to ask the health provider questions, were not given privacy and were not encouraged to have a support person present with them. Only in very rare cases did a health worker physically abuse or yell at his or her patient.

This study is among the first to quantify the prevalence of disrespect and abuse during labor and delivery in Malawi through direct observations. In Malawi, efforts are currently being made to improve how women are treated but there are still some issues that remain to be addressed.

## Background

It is known that most maternal deaths can be prevented with appropriate care that includes access to skilled birth attendants and quality emergency obstetric and newborn care (EmOC) [1]. While the availability of quality clinical obstetric services is a key determinant of the delivery outcome of a mother and her newborn, a pregnant woman and/or her support person must first take action and seek care in a health facility to receive care by skilled birth attendants. However, there are well-described demand and supply-side barriers that can prevent this from occurring [2].

Disrespect and abuse during childbirth can affect both a woman’s decision to seek care, for example, fearing ill-treatment may prevent her from seeking care at a health facility as well as quality of care she receives including being mistreated or cared for inappropriately [3]. In 2010, Bowser and Hill conducted a landmark landscape analysis of disrespect and abuse in facility-based childbirth where they reviewed evidence related to the definition, scope, contributors and impact of disrespect and abuse in childbirth. The results led to the development of seven categories of disrespect and abuse [3]. Bohren et al. (2015) subsequently developed a typology of mistreatment of women during labor in which they defined first, second, and third order themes based on their mixed methods systematic review of evidence on the mistreatment of women during childbirth in health facilities [4]. The White Ribbon Alliance led a multi-sectoral collaboration to produce a consensus document--the Respectful Maternity Care Charter: The Universal Rights of Childbearing Women [5]. These rights are described as universal and are therefore relevant to all pregnant women around the world but are of particular interest in low resource settings where women may have less choice about where to deliver, less power to demand respectful care, and less legal recourse to address and challenge disrespectful treatment. According to the World Health Organization’s statement on the prevention and elimination of disrespect and abuse during facility-based delivery, “…pregnant women have a right to be equal in dignity, to be free to seek, receive and impart information, to be free from discrimination, and to enjoy the highest attainable standard of physical and mental health, including sexual and reproductive health” [5]. While disrespect and abuse during delivery does not necessarily mean that respectful care was provided, it does mean that the fundamental human right of women to receive the highest attainable standard of care was violated [6, 7].

### Mother- friendly care in Malawi

In Malawi, the ethical and respectful treatment of maternity clients is also an important issue that has not been quantitatively measured. Even without quantitative evidence, efforts have been made to improve the experience of care during labor and delivery. For example, The Integrated Maternal and Newborn Care Training Manual for Malawi that is part of the training curriculum for health workers who provide labor and delivery services contains modules that cover the knowledge, skills and attitudes required for skilled birth attendants to provide emergency obstetric and newborn care [8]. Module 8 of this manual (Management of Second Stage of Labor) describes “Mother and Family Friendly Care Guiding Principles” and includes mother friendly care actions that should be taken during the management of labor. This training manual was developed and rolled out in 2007 and finalized in 2009 after being pre-tested. Any health worker who provides labor and delivery services and who graduated after 2008 will have received training that includes mother friendly care actions. Table 1 provides examples of the mother friendly care actions that are included in the training manual that reflect respectful maternity care and the corresponding category of disrespect and abuse as per the Bowser and Hill (2010) landscape analysis.

**Table 1:** Mother friendly care actions listed in the integrated maternal and newborn care training manual (Ministry of Health, Malawi)

MOTHER-FRIENDLY CARE ACTIONS	Corresponding category of disrespect and abuse that is addressed by the action
• Kind and supportive care	Non-dignified care
• Body language that shows kindness (address woman by name, look into the woman’s eyes, uses a respectful tone of voice, smiles when appropriate)	Non-dignified care
• Privacy	Non-confidential care
• Clean and attractive facility	Non-dignified care
• Permit cultural practices that are not harmful	Non-dignified care/Abandonment or denial of care
• Explain every procedure	Non-consented care
• Episiotomy only if indicated	Non-consented care
• Choice of position for delivery	Non-dignified care
• Give woman and family friendly care. Explain what is happening to the woman and family after each evaluation. Teach the woman and companion how to support the woman in labour: o Urinate every 2 h o Drink fluids every 1 h or more often o Eat lightly o Have a birth support/guardian person present o Talk to the woman: give emotional support and educate her about what is happening o Use comfortable positions for labour (walking, sitting, side-lying)	Non-consented care/ Abandonment or denial of care
• Postpartum o No restriction on family members o No separation of mother and baby	Abandonment or denial of care

Table adapted directly from Module 8 of the Participants Manual in Integrated Maternal and Newborn Care. (2009). Malawi Ministry of Health, Reproductive Health Unit

There is limited quantitative evidence available on the observed treatment of women during labor and delivery in resource poor settings. Rosen et al. (2015) presented quantitative results from more than 2000 observations of labor and delivery in five countries (Ethiopia, Kenya, Madagascar, Rwanda and Tanzania) and found that women were generally treated with dignity but that many women were subject to poor interactions with providers and were not well-informed about their care. They also documented physical and verbal abuse [9]. In Tanzania, quantitative results from both observations of labor and delivery and from postpartum client interviews showed that 70% of women interviewed during follow-up interviews reported experiencing any disrespect and abuse [10]. In Kenya, 20% of women who participated in exit interviews reported any form of D&A, including non-confidential care (8.5%); non-dignified care (18.0%); neglect or abandonment (14.3%); non-consensual care (4.3%); physical abuse (4.2%); and detainment for non-payment of fees (8.1%) [11]. In Ethiopia, 71.8% of women interviewed reported experiencing one or more categories of disrespect and abuse, with all women who delivered in hospitals reporting the violation of the right to information, informed consent, and choice/preference of birth position [12]. However, this type of evidence of D&A is currently unavailable in Malawi. Therefore, the objective of this research is to measure the prevalence of disrespect and abuse during labor and delivery through the secondary analysis of direct clinical observations and to describe the association between the observation of D&A items with the place of delivery and client background characteristics.

## Methods

### Study design, setting and sample

This descriptive secondary analysis of respectful maternity care was conducted by using existing data from direct clinical observations of labor and delivery conducted during the second round of data collection from the Helping Babies Breathe (HBB) evaluation in August 2013. Data were collected from 40 health facilities (28 hospitals and 12 health centers) that were chosen based on high delivery volume from 27 out of the 28 districts in Malawi. Health workers were selected for observation based on availability at the time of data collection, willingness to be observed, and status as a skilled birth attendant as per local classification (doctor, medical assistant, clinical officer, registered midwife, enrolled nurse midwife or nurse midwife technician).

A total of 20 clinical observers were trained for one week on the use of a labor and delivery observation (L&D) checklist, further described below. They practiced observations using the L&D checklist until the inter-rater reliability across all observers was at least 80% as per the Clinical Observer Training Guidelines developed by the Maternal Child Health Integrated Program (MCHIP) [13]. Information on the timing of the observation of the item is being presented because the disrespect and abuse items are observed in different stages of labor (1st or 3rd) or as part of the overall outcome of the observation (so would apply to all women observed). Therefore more data is available on items that occur in the third stage of labor or the outcome than in the first stage of labor.

### Data collection

The main data collection tool used to generate data for this analysis was an L&D observation checklist. This checklist adheres to the World Health Organization’s guidelines on Managing Complications in Pregnancy and Childbirth and was adapted from the USAID-funded Prevention of Postpartum Hemorrhage Initiative (POPPHI) Project labor and delivery checklist [14]. This checklist was designed to capture information about whether health workers performed key evidence-based interventions during the labor and delivery period and was divided into four main sections: initial client assessment; intermittent observation of the first stage of labor; continuous observation of the second and third stage of labor; immediate newborn and postpartum care and outcome review and documentation. While the checklist included some items reflecting interpersonal communication between the health provider and the patient, several aspects of care related to the seven categories of disrespect and abuse that were absent in the original checklist were added. The selection of the additional items was based on a compendium of proposed indicators developed by the Maternal Child Health Integrated Program (MCHIP). These indicators were reviewed by members of the technical team at MCHIP who specialize in respectful maternity care and monitoring and evaluation as well as members of the Reproductive Health Department of Malawi’s Ministry of Health. Table 2 shows the D&A items used in the analysis and the timing of the observation during labor and delivery.

**Table 2:** D&A items and timing of observation during labor and delivery

Direct clinical observation item	Stage of labor
Non dignified care	
Did not respectfully greet pregnant woman	First
Shouted, insulted or threatened the woman during labor or after	Applies to all observations
Non consented care	
Manual exploration of uterus after delivery when unindicated	Applies to all observations
Used episiotomy (without indication)	Applies to all observations
Did not ask woman (and support person) if she has any questions	First
Did not ask client if there are any other problems the client is concerned about	First
Did not explain procedures to woman (support person) before proceeding	First
Did not inform the woman what will happen before conducting the vaginal examination	First
Did not inform pregnant woman of findings	First
Did not explain what will happen in labor to woman (support person) at least once	First
Did not explains procedures to woman (support person) before proceeding	First
Provider did not give at least one update on status and progress of labor	Third
Non confidential care	
Woman did not have audio and visual privacy	First
Provider did not drape woman (one drape under buttocks, one over abdomen)	First
Woman did not have her own bed	First
Provider did not use curtains or other visual barriers to protect woman during exams, births, procedures	First
Abandonment or denial of care	
Provider did not encourage the woman to have a support person present during labor and delivery	First
Provider did not encourage woman to consume fluids/food during labor at least once	First
Provider did not encourage or assist woman to ambulate and assume different positions during labor at least once	First
Provider did not ask woman which position she would like to deliver in	First
Support person or companion for mother was not present at birth	Third
If support person was not present at birth: Support person was restricted from being present	Third
Woman requested some pain relief for her pain but was not given anything	Third
Woman was not allowed to deliver in her preferred birthing position (if she had a preferred position)	Third
Mother and newborn were not kept in same room after delivery (rooming-in)	Third
Physical abuse	
Provider slapped, hit or pinched the woman during labor or after	Applies to all observations

Clinical observers were required to observe an average of five deliveries per day over 12 days in order to be able to make at least 50 observations of labor and delivery starting at the third stage of labor and five deliveries starting at the first stage of labor.

### Data analysis

Descriptive analysis was performed using Stata statistical software (Version 13, College Station, Texas USA). Exploratory data analyses were conducted to examine the extent of missing data and distribution of the outcome and explanatory variables in the sample. Descriptive statistics including frequencies and proportions were calculated to describe health worker characteristics and overall performance. Bivariate analysis that accounted for clustering by health provider was conducted using logistic regression models (where the relationship between the odds of the observation of a D&A item was assessed against facility type (hospital or health center) and client-level factors—woman’s age, parity, and HIV status.

## Results

A total of 2109 direct clinical observations were conducted, of which 208 began at first stage of labor and the remaining 1901 commenced at the third stage of labor. Data on potentially harmful practices and on maternal and newborn outcomes was recorded for all observations. Nearly three quarters (71.3%) of the delivery observations were conducted in a hospital while the rest were made in health centers. As presented in Table 3, the median age of the respondents was 23 years of age; their median parity was one pregnancy; and the proportion of observed women who were HIV positive was 6.5%.

**Table 3:** Characteristics of observed women observed during labor and delivery in 40 health facilities in Malawi in 2013

Characteristics of observed women (*n* = 2109)	
Median age in years (Interquartile range)	23 (IQR: 9)
Median parity (Interquartile range)	1.0 (IQR: 3)
Proportion of women who were HIV positive (%, n)	6.5% (111)^a^

^a^Denominator = 1718; data on HIV status missing for *n* = 391 missing

### Descriptive analysis of D&a items for all labor and delivery observations

The overall frequency that a D&A-related item occurred ranged from .09% (for manual exploration of the uterus after delivery when unindicated) to 93.7% (for the health provider not asking the woman in which position she wanted to deliver) (Table 4). Under non-dignified care, 13.9% of women were not greeted respectfully and 1.9% of women were shouted at, insulted, or threatened during labor or after. Non-consented care, including unindicated manual exploration of the uterus after delivery and episiotomy were rarely performed (.09% and.50%, respectively). However, women were not encouraged to have a support person, were not asked if they had any questions, and were not asked if they had any other problems they were concerned about on a much more frequent basis (83.2%, 73.1% and 73.9%, respectively). Under non-confidential care, more than half of women (58.2%) did not have audio and visual privacy. Under abandonment/denial of care, one third of women (33.7%) observed were not encouraged by the provider to consume fluids/food during labor at least once and most women were not asked about the position in which they wanted to deliver (93.7%). Less than 1 % (.20%) of women were slapped, hit or pinched by the provider during labor or after.

**Table 4:** Descriptive D&A results for all L&D observations from 40 health facilities in Malawi in 2013

Direct clinical observation item	Number of observations	Number of occurrences	Frequency of occurrence
Non dignified care			
Did not respectfully greet pregnant woman^a^	208	29	13.9%
Shouted, insulted or threatened the woman during labor or after^c^	2109	41	1.9%
Non consented care			
Manual exploration of uterus after delivery when unindicated^c^	2109	2	0.09%
Used episiotomy (without indication)^c^	208	1	0.50%
Did not ask woman (and support person) if she has any questions^a^	208	152	73.1%
Did not ask client if there are any other problems the client is concerned about^a^	203	150	73.9%
Did not explain procedures to woman (support person) before proceeding^a^	205	35	17.1%
Did not inform the woman what will happen before conducting the vaginal examination^a^	205	42	20.5%
Did not inform pregnant woman of findings^a^	200	20	10.0%
Did not explain what will happen in labor to woman (support person) at least once^a^	208	43	20.7%
Did not explain procedures to woman (support person) before proceeding^a^	208	32	15.4%
Provider did not give at least one update on status and progress of labor^b^	2052	249	12.1%
Non confidential care			
Woman did not have audio and visual privacy during initial client assessment^a^	208	121	58.2%
Provider did not drape woman (one drape under buttocks, one over abdomen)^a^	208	152	73.1%
Woman did not have her own bed^a^	208	5	2.4%
Provider did not use curtains or other visual barriers to protect woman during exams, births, procedures^a^	206	54	26.2%
Abandonment or denial of care			
Did not encourage the woman to have a support person present during labor and delivery^a^	208	173	83.2%
Provider did not encourage woman to consume fluids/food during labor at least once^a^	208	70	33.7%
Provider did not encourage or assist woman to ambulate and assume different positions during labor at least once^a^	208	58	27.9%
Provider did not ask woman which position she would like to deliver in^a^	207	194	93.7%
Support person or companion for mother was not present at birth^b^	2071	1818	87.8%
If support person was not present at birth: Support person was restricted from being present^b^	1818	210	11.6%
Woman requested some pain relief for her pain but was not given anything^b^	118	66	55.9%
Woman was not allowed to deliver in her preferred birthing position (if she had a preferred position)^b^	273	36	13.2%
Mother and newborn were not kept in same room after delivery (rooming-in)^b^	1722	213	12.4%
Physical abuse			
Provider slapped, hit or pinched the woman during labor or after^c^	2109	4	0.20%

^a^applies to first stage of labor

^b^applies to third stage of labor

^c^applies to all observations

### Relationship between D&a items, facility type and client background characteristics

The results of the bivariate analysis presented in Table 5 do not show a consistent difference in the treatment of women based on whether or not they delivered in a hospital or health center. For example, the odds of a health provider shouting at a woman were 81% lower in health centers when compared to hospitals (OR: 0.19; CI: 0.59–0.62) but the odds of a health provider not asking the woman if there are other problems she is concerned about during the initial client assessment were 2.4 times higher in a health center when compared to a hospital (OR: 2.40; CI: 1.06–5.40). The odds of not having a support person present was 2.6 times higher in health centers (OR: 2.61, CI: 1.82–3.73) and the likelihood of a support person being restricted from being present was higher in health centers than in hospitals (OR: 1.62, CI: 1.21–2.19).

**Table 5:** Results of bivariate analysis of D&A items with facility type and client’s age, parity and HIV status

	Unadjusted Odds Ratio							
RMC Item	Facility type^a^	*p*-value	Age	*p*-value	Parity	*p*-value	HIV status^b^	*p*-value
Non Dignified Care								
Does not respectfully greet woman	2.14	0.10	1.01	0.72	0.94	0.61	1.21	0.79
Shout, insult or threaten the woman during labor or after	0.19	0.006	0.99	0.55	0.91	0.36	1.46	0.53
Non-consented care								
Does not ask woman (and support person) if she has any questions	0.51	0.12	0.99	0.80	0.94	0.61	0.81	0.68
Does not ask client if there are any other problems the client is concerned about	2.40	0.036	0.98	0.42	1.11	0.26	0.38	0.04
Does not explain procedures to woman (support person) before proceeding	1.29	0.52	0.99	0.90	1.02	0.81	0.99	0.90
Does not inform the woman what will happen before conducting the vaginal exam	0.85	0.79	0.96	0.19	1.01	0.91	0.74	0.64
Does not inform pregnant woman of findings	1.15	0.81	1.00	0.99	1.04	0.79	0.46	0.46
Does not explain what will happen in labor to woman (support person) at least once	1.41	0.52	0.96	0.22	0.85	0.09	1.05	0.93
Does not explain procedures to woman (support person) before proceeding	1.15	0.83	0.97	0.34	0.87	0.15	0.62	0.56
Provider does not give at least one update on status and progress of labor	0.64	0.006	1.01	0.18	0.95	0.24	1.26	0.43
Non-confidential care								
Woman does not have audio and visual privacy (during initial client assessment)	0.91	0.80	1.00	0.98	1.10	0.36	0.34	0.05
Provider does not drape woman (one drape under buttocks, one over abdomen)	0.98	0.96	0.99	0.66	0.95	0.60	1.13	0.84
Woman does not have her own bed	0.50	0.56	0.85	<0.001	0.64	0.24	n/a^c^	n/a
Provider does not use curtains or other visual barriers to protect woman during exams, births, procedures	2.99	0.020	1.00	0.88	1.04	0.60	0.38	0.23
Abandonment or denial of care								
Does not encourage the woman to have a support person present during labor and delivery	1.43	0.52	1.00	0.99	1.00	0.98	0.64	0.50
Does not encourage woman to consume fluids/food during labor at least once	0.24	0.001	1.00	0.86	0.92	0.40	1.59	0.40
Does not encourage or assist woman to ambulate and assume different positions during labor at least once	0.24	0.004	1.03	0.23	1.01	0.89	0.65	0.54
Provider does not ask woman which position she would like to deliver in	2.54	0.28	0.98	0.71	0.91	0.66	0.17	0.010
Support person or companion for mother is not present at birth	2.61	0.001	1.02	0.11	1.26	<0.001	2.00	0.06
If support person was not present at birth: Support person was restricted from being present	1.62	0.18	1.00	0.69	0.94	0.20	0.56	0.16
Woman requested some pain relief for her pain but was not given anything	0.29	0.24	0.96	0.16	0.81	0.06	n/a^c^	n/a
Woman was not allowed to deliver in her preferred birthing position (if she had a preferred position)	0.53	0.26	1.03	0.07	1.06	0.56	0.77	0.68
Mother and newborn were not kept in same room after delivery (rooming-in)	1.44	0.16	0.98	0.12	0.96	0.32	0.89	0.70

^a^Facility type coded 0 for hospital and 1 for health center

^b^HIV status coded 0 for HIV- and 1 for HIV+

^c^n/a: The number of HIV positive women in the subset of data who requested pain relief and did not have their own beds were not computed due to few observations in cells for these two RMC items

The bivariate analysis between age of the woman and D&A items showed even fewer associations; the only statistically significant difference seen was between age and whether the woman had her own bed, where the odds of not having her own bed was 15% lower as the age of the woman increased (OR: .85, CI: 0.79–0.92). There was also an association between the presence of a support person and parity, where there was a 26% higher odds of not having a support person present as the woman’s parity increased (OR: 1.26, CI: 1.13–1.41). For the prevalence of D&A items and HIV status, women who were HIV+ had significantly lower odds of not having audio and visual privacy (OR: 0.34, CI: 0.12–0.97), of not being asked about her preferred delivery position (OR: 0.17, CI: 0.05–0.65) and of not being asked if she has any other problems she is concerned about (OR .38, CI: 0.15–0.96).

## Discussion

The goals of this analysis were to provide an estimate of the prevalence of disrespect and abuse during childbirth in Malawi through the secondary analysis of labor and delivery observations and to assess the association of selected client-level characteristics with disrespect and abuse. More specifically, the hypothesized dimensions were based on observable dimensions of the Bowser and Hill (2010) landscape analysis of disrespect and abuse during facility-based childbirth, including physical abuse, non-consented care, and non-dignified care [3].

According to the Malawi DHS 2015/16, 94.8% of pregnant women in the country receive antenatal care from a skilled attendant and 89.8% deliver with a skilled birth attendant, indicating high access to health facilities [15]. However, issues still remain with the quality of services and the experience of care during those deliveries. A qualitative study in rural Malawi found that staff in the labor and delivery wards did not communicate with patients and kept women waiting for their examinations. This study also cited findings from the Malawi Obstetric Quality of Care assessment that found that one of the major constraints to accessing maternal health services was rudeness of health workers [16]. In the present analysis of L&D observations, the waiting time for the client to initially receive care was not recorded but communication between the provider and the client was observed. Communication was found to be high in some aspects (e.g. informing the woman of findings from exams) but lower in other aspects (e.g. asking the woman if she has any questions) and the majority of clients were greeted respectfully. However, the finding of deficiencies in the quality of interpersonal communication from the health worker to the patient is not surprising, as problems with communication between health workers and patients have previously been reported in the media in Malawi [17].

Another study in three districts in Malawi reported that health workers created barriers to care-seeking by being unwilling to assist pregnant women, beating women in labor, acting rudely, performing operations when drunk, using abusive language, discriminating against poor women, delaying treatment and not providing privacy [18]. Discrimination against women was difficult to observe through direct clinical observations in the present analysis but the results of the bivariate analysis showed that there were statistically significant differences between having audio/visual privacy and being asked about a preferred delivery position between women based on HIV status. More specifically, HIV-positive women were more likely to have audio and visual privacy and to be asked about their preferred delivery position or any other problems they may have been concerned about. The findings differ somewhat with results from Sando et al. (2014), where they assessed self-reported disrespect and abuse during childbirth in Tanzania and found no reported differences in the likelihood of reporting D&A between HIV positive and HIV negative women [19]. However, the data collection methods used between that study and the present analysis differ.

Women in the present analysis were not generally given a choice of birthing position, but this could potentially be because health providers may not have been comfortable assisting women in positions other than the orthodox supine position. In Tanzania, it was found that labor position was not a crucial factor in deciding where women chose to deliver so while these findings are important, it may not be the ultimate deterrent of facility-based births [20]. The present results showed low frequencies of audio and visual privacy and the lack of encouragement by health providers to have a support person present. However, the restriction of a support person happened in only a small number of cases. This finding suggests that facility infrastructure may in fact result in the occurrence of some D&A items due to lack of space in the facility rather than the direct behavior of the health worker. However, further research will need to be conducted to understand how structural level deficiencies relate to provider-level behaviors.

A qualitative study in Malawi by Kumbani et al. (2013) found that the major concern for recently delivered women was poor staff attitudes. Women reported that health workers shouted at them and even threatened to beat them if they created problems during delivery. Other participants reported meeting rude health workers that treated them harshly during labor and delivery and did not treat them like human beings. The women in that study perceived poor care when they were shouted at, ignored, there were delays in receiving care, or they were not informed of findings. They did not complain about the technical quality of care [21]. However, the results from the present study using labor and delivery observations rather than qualitative data showed that most women were informed of findings and that shouting and threatening or physically abusing women during labor or after did not occur frequently. Lack of privacy was an issue in this study and another study in Malawi also found that privacy was difficult to achieve, that some beds did not have curtains and that many people had access to the ward [16]. This is an important result because in Malawi, being respected, respectfully greeted, informed of findings, and having confidentiality and privacy are associated with good quality of care [21].

While the Ministry of Health of Malawi has been proactive in addressing issues of disrespect and abuse during labor and delivery through the Malawi National Reproductive Health Service Delivery Guidelines [22], the results from this analysis of labor and delivery observations showed that some women are still exposed to some level of disrespect and abuse during delivery. The Ministry of Health updated and finalized the Reproductive Health standards in January 2017 and has included disrespect and abuse through performance standards to be assessed and evaluated at the facility and national levels by national assessors. This effort may further reduce D&A because facilities that are recognized as ‘Centers of Excellence’ will be expected to promote positive delivery experiences [22].

Additional work should also be done to develop and validate instruments to measure facility-based D&A during childbirth through clinical observations so that changes in prevalence of these items can be monitored in a standardized way across time.

### Limitations

Because of limitations in what can be directly observed, observations reflecting the entire Bowser and Hill categories of D&A could not be included in the direct clinical observation tool. There are currently no validated instruments available to measure D&A during childbirth in health facilities using clinical observations, which may create challenges if studies use different operational definitions of D&A and make attempts to compare results. The clinical observation ended after immediate newborn care so it is not possible to know how the client was treated after the labor and delivery observation ended. Detention for lack of payment was not included in the observation checklist because services are offered for free in the government-run study facilities. Discrimination is also a challenging concept to observe unless the health workers provide verbal cues.

Regarding the data itself, the selection of health workers and labor and delivery cases to observe was a non-probability sample. The lack of randomization of health workers may have introduced selection bias since health workers who agreed to participate may have been different from those who refused. As was previously mentioned, data related to disrespect and abuse during labor and delivery has generally been collected through qualitative data collection methods. Information about the client’s expectations of the health worker during labor and delivery (and vice versa) in Malawi was not included in the direct clinical observation tool. Also, while negative associations between the quality of health worker-patient communication and patient literacy and socioeconomic status have been documented [18], this information was not available in this study.

The Hawthorne effect may also be a factor in observing health worker performance, especially related to disrespect and abuse. The method of direct clinical observations could influence the health worker’s performance (either negatively or positively). These data were also collected in government-run health facilities so information was not available on whether the treatment by private health providers or traditional birth attendants differed from these government health providers. It is also possible that certain items that were more overt under observation (e.g. physical and verbal abuse) were subject to a higher level of the Hawthorne effect, but this was not measured.

## Conclusion

The results showed that overall, women were generally greeted respectfully and were informed of procedures, but women were not encouraged to ask questions, were not given privacy, and were not encouraged to have a support person with them. The prevalence of several D&A items was also found to be lower amongst HIV-infected women who delivered in these health facilities than HIV-positive women. The findings of this research provide evidence of some aspects of D&A through direct observations but further research should be conducted to understand health worker-level barriers and facilitators to reducing disrespect and abuse as well as using additional methods to measure D&A in Malawi.

## Abbreviations

D&A: Disrespect and abuse
EmOC: Emergency obstetric and newborn care
HBB: Helping Babies Breathe
L&D: Labor and delivery
MCHIP: Maternal Child Health Integrated Program
POPPHI: Prevention of Postpartum Hemorrhage Initiative
RMC: Respectful maternity care
USAID: United States Agency for International Development
WHO: World Health Organization
